# AGHE PROGRAM OF MERIT: GERONTOLOGY DEGREE PROGRAMS, MINORS, AND CERTIFICATES

**DOI:** 10.1093/geroni/igz038.2115

**Published:** 2019-11-08

**Authors:** Shannon D Mathews, Pamela Elfenbein

**Affiliations:** 1 Winston Salem State University, Winston Salem, North Carolina, United States; 2 University of North Georgia, Oakwood, Georgia, United States

## Abstract

The Executive Committee of the Academy for Gerontology in Higher Education (AGHE), formerly known as the Association for Gerontology in Higher Education, approved a proposal to establish and implement a voluntary program of evaluation known as the Program of Merit (POM). The POM designation provides gerontology programs with an AGHE “stamp of approval,” which can be used to verify program quality to administrators, to lobby for additional resources to maintain a quality program, to market the program, and to recruit prospective students into the program. In 2014, AGHE Gerontology Competencies for Undergraduate and Graduate Education© were established and have been integrated into this process. This session will outline the Program of Merit process and present the opportunities for gerontology program leaders to advance gerontology education.

